# Our Journal

**Published:** 1856-01

**Authors:** 


### OUR JOURNAL.

The present number, the first of the third volume, has been delayed, from the inability on the part of the publishers, to obtain a good quality of paper at the time.

It will be seen that there is an unusual amount of original matter in this number, for which we feel indebted to our contributors. As we design an improvement in this over the former volumes, we trust our kind friends and professional brethren will continue to furnish the results of their observation and experience from time to time. We are aware that it is sometimes difficult to preserve notes of cases, and that there is little time for making them up for publication, and hence a reluctance to report. But every medical man should consider himself under heavy obligations to medical science and humanity, to preserve the record of every case, the phenomena of which are peculiar, and report them for the good ot others. When we receive reports from a medical man from time to time, we take it for granted that *that* man is deeply interested in advancement in his profession, and also that he is destined to place himself in a prominent relation to *that* profession. Not that the converse is always true either, but yet we have no means of knowing this unless the affirmative is observed.

Two volumes have been published, the last a decided improvement upon the first. The coming volume, we hope will, as before said, be still an improvement. Our readers are, perhaps, not aware of the labors, cares, and annoyances experienced in the editorial management of a work of this kind. Although only published every alternate month, yet the task is quite onerous enough for one individual, provided his whole time and labor be devoted to its pages. But to the cares, labors, and responsibilities of a practice and our college duties, we have had superadded the editorial charge of this periodical. This, together with our inexperience, will, we trust, sufficiently apologise for many defects and errors which have appeared in previous numbers.

We have labored in the past to make our Journal just such as an enlightened profession would require and demand, and we rejoice that we have been favored by its smiles of endorsement and encouragement. This last cheers us with the hope, which is strong within us, that it will be fostered and cherished, and that liberal aid will be afforded us in the enterprise in order to its maintenance. This is all we asked or expected, in return for our efforts in this work, and it is all we crave still.

In our efforts to close up the accounts for subscription, we employed a third person to make out bills and enclose them to delinquents. In some instances, errors have occurred, which, when discovered, have been promptly corrected. This statement, we hope, will be satisfactory to all who know, as all should, that errors will creep in, when our business is with so many individuals and all has to be transacted through the mail. Those who are so illiberal or ignorant of this fact, and become captious and uncourteous in their address when an error of this kind may have occurred with them, have the assurance that such mistakes will not occur again, if it can be avoided.

We beg those who know themselves indebted for subscription to forward as soon as possible, as we are much in need of the amount due from each. We cannot afford to lay out of our own means, and therefore we entreat those yet in arrears to take this into consideration.

☞ In future, all communications for publication will be addressed to Drs. M'Gugin & Allen; and all letters on business or subscription must be sent to Dr. J. C. Hughes. By this arrangement the *editorial department* will be kept separate from the business matters of the Journal.

